# Is Single Gloving Still Acceptable? Investigation and Evaluation of Damages on Sterile Latex Gloves in General Surgery

**DOI:** 10.3390/jcm10173887

**Published:** 2021-08-29

**Authors:** Andreas Enz, Imad Kamaleddine, Justus Groß, Clemens Schafmayer, Emad Alwafai, Larissa Sievers, Wolfram Mittelmeier, Annett Klinder

**Affiliations:** 1 Orthopaedic Clinic and Policlinic, University Medical Centre Rostock, 18057 Rostock, Germany; larissasievers@googlemail.com (L.S.); wolfram.mittelmeier@med.uni-rostock.de (W.M.); annett.klinder@med.uni-rostock.de (A.K.); 2 Department of General, Visceral, Vascular and Transplant Surgery, Surgical Clinic and Polyclinic of University Medical Centre Rostock, 18057 Rostock, Germany; imad.kamaleddine@med.uni-rostock.de (I.K.); Justus.Gross@med.uni-rostock.de (J.G.); clemens.schafmayer@med.uni-rostock.de (C.S.); Emad.Alwafai@med.uni-rostock.de (E.A.)

**Keywords:** microperforations, EN455, ASTM D5151-19, surgical side infections, general surgery

## Abstract

(1) Background: The sterile latex surgical glove is an important part of protecting both the patient and the surgical team from infections. However, mechanical stress can damage the integrity of the glove material and thus may lead to infections. (2) Method: A total of 896 gloves from 448 surgeries were tested and evaluated by the water tightening test according to EN455 and ASTM D5151-19. (3) Results: From 448 surgeries, 18.8% of the interventions showed glove damage. In vascular surgery, gloves were damaged in 20.8%, in thoracic surgery 9.1%, in laparoscopic interventions 21.7%, in the subgroup hernia surgeries (TAPP) 17.6% and in open interventions 17.6%. A total of 101 damages were found on 896 gloves; one glove could have several damages. During vascular surgery, 60% of the damages were on the subordinated hand of the surgeon, and 73.3% of the damages had a size of 1 mm. In laparoscopic procedures, the subordinated hand was also more frequently affected (61.3%) than the dominant hand; 64.5% of the damages were 1 mm in size. In the hernia surgery subgroup (TAPP), no damage was larger than 1 mm; 66.7% were in the subordinated hand area. The duration of surgery had no influence on the lesion rate. (4) Conclusion: The damage rate in low impact procedures is high and represents an underestimated problem in soft tissue surgery. The use of single gloving can therefore lead to the risk of infection. EN455 and ASTM D5151-19 does not take into consideration the risk of intraoperative lesions. Double gloving and glove change algorithms should be established.

## 1. Introduction

Since the 1890s, surgical gloves have been worn to reduce or prevent wound infections in surgical interventions [1] and thus laid the basis for modern surgery [2]. In current hygiene concepts, the sterile latex surgical glove still plays an important role, as it protects both the patient and the surgical team from infections [3]. However, this medical device made of thin latex is subject to mechanical stress [4], which damages the integrity of the glove and thus its protective function [5]. This could lead to sepsis or other infections, some of the most serious complications of surgery and can have both personal consequences for the patient and economic consequences for the health systems [6]. On the other hand, these damages violate the integrity of the gloves and allow the transmission of pathogens to the medical staff, with far-reaching consequences [7].

Surgical gloves are used in many medical fields, including orthopaedics and surgery [8]. The production of gloves is subject to standards like the European committee of standardization (CEN) European norm (EN) 455 [9] or the American Society for Testing and Materials (ASTM) D3577-19 [10] and D5151-19 [11], which describe almost identical specifications. The standards specify the water tightening test for the detection of holes. One litre of water is put for 2–3 min into the glove to be tested, which is fixed in a defined holding device. If no water leaks, the glove is considered to be waterproof. Differences are in the acceptable quality limit (AQL), which was reduced to 0.65 in the latest edition of EN455 in 2020; the AMST D3577 limit is 1.5 [9,10,11]. An AQL of 1.5 means that for 125 tested latex gloves, five gloves can be damaged without having to discard the production lot. Thus, gloves are subject to a clearly poorer AQL than, for example, latex condoms (AQL 0.25) [12]. Mechanical stresses on gloves such as those occurring during surgical interventions are not taken into account in the applicable standards. In orthopaedic surgery, due to the handling of medical devices and the force used to implant these devices, double gloving (wearing two gloves on top of each other) has been a practice for many years [13]. In soft tissue surgery, this is recommended but was not considered necessary due to the absence of sharp-edged tissue or implants and less stress on the gloves. However, this problem has received little scientific and clinical attention to date. The aim of this study was to document and demonstrate the vulnerability of gloves to perforation after various surgical procedures, to develop glove changing algorithms and to highlight the necessity of double gloving. A total of 896 gloves from 448 surgeries were tested and evaluated by the water tightening test according to EN455 and ASTM D5151-19.

## 2. Materials and Methods

### 2.1. Clinic and Surgeries

Gloves from 448 surgeries including laparoscopic and open surgery, vascular and thoracic surgery were collected from January to September 2020 and examined.

### 2.2. Gloves

In total, 896 surgical gloves from 448 surgeries were examined. The total number of used gloves, the number of gloves per surgery and the type and the duration of the surgery were documented. All surgeries were performed with one pair of sterile powder-free latex gloves for single use. ProtexisTM, Cardinal Health, Dublin, Ohio, OH, USA (AQL 0.65); Biogel Eclipse, Mölnlycke Health Care, Gothenburg, Sweden (AQL 0.65) and Sempermed supreme, Sempermed/Semperit, Vienna, Austria (AQL 0.65) were investigated. All gloves used were included in the study; there were no exclusion criteria.

### 2.3. Method of Investigation

The gloves of the leading surgeon were collected and packed at the end of each operation, and the data relevant for evaluation were documented. The examination was carried out in the laboratory. As a control, the influence of glove undressing was tested on 50 gloves without surgery. Fifty gloves without surgery were tested after postage (3 days duration). A total of 50 pairs of gloves of each manufacturer were tested for lesions in the unused condition (batch testing). The evaluation was performed according to EN 455—Medical gloves for single use part 1, method for testing for freedom from holes with water tightening test. Only gloves that failed the water tightening test were further investigated. The localization of the damage was determined, the size and dimension were measured with a plastic goniometer (Kirchner & Wilhelm GmbH & Co. KG, Asperg, Germany) and the lesion configuration was recorded with microscopes (Laser Scanning Microscope VK-S1100 and Digital Microscope VHX-6000, Keyence, Germany). 

### 2.4. Statistics

The collected data were analysed using SPSS Statistics Package Version 22 (IBM Corp., New York, NY, USA). Descriptive statistics were calculated for continuous and categorical variables. Continuous variables are displayed as mean values and standard deviations (SD) as well as median and range, as most of the data were not normally distributed. Categorical factors are shown as frequency (*n*) with percentages in brackets. Testing for differences between different types of surgeries of categorical factors was performed using Pearson’s chi-square test. Testing for differences in continuous variables between different types of surgeries was performed using the Kruskal–Wallis test. The significance level was set at *p* < 0.05.

### 2.5. Ethics Vote and Data Privacy

Ethics approval for the study was granted by the local ethics committee (A2020-0236), and data protection requirements were observed.

## 3. Results

### 3.1. General Data and Examined Surgical Treatment

A total of 448 surgeries were included: 488 gloves from 244 open surgeries (OS), 106 gloves from 53 vascular interventions (VS), 22 gloves from 11 thoracic surgeries (TS), 212 gloves from 106 laparoscopic procedures (LS) and 68 gloves from 34 laparoscopic hernia interventions (TAPP). In total, 896 gloves, were collected and examined. All surgeons involved were right-handed. Descriptive data are presented in Table 1. The types of surgery that were performed within the defined groups are specified in Table 2.

### 3.2. Gloves and Operating Time

The duration of use was between 2 and 397 min. A correlation between surgery time and number of glove damages could not be confirmed (correlation coefficient r = 0.053, *p* = 0.262). The gloves were worn for an average of 95.0 min (±78.0) during open surgery, 100.4 (±55.9) min for vascular procedures, 100.7 (±61.6) min for thoracic surgery, 83.7 (±53.3) min for laparoscopy and 58.1 (±27.0) min in the TAPP group (see Table 1). Overall, half of the surgeries were carried out in less than 70 min, as the median of wear time for all 448 surgeries was 70 min. When analysed with regard to risk stratification, as published by Partecke et al. [14], 62.9% of surgeries lasted 0–90 min, 20.8% 91–150 min and 16.3% >150 min.

### 3.3. Comparison of Intraoperative Lesions

All detected damages remained unnoticed intraoperatively. The five groups of surgery, namely OS, VS, TS, LS and TAPP, are compared in Table 3. A total of 106 damages were found; one glove could have several damages. A total of 18.8% of the interventions showed glove damage. In VS, gloves were damaged in 20.8% of surgeries, in TS 9.1%, in LS 21.7%, in the subgroup hernia surgeries (TAPP) 17.6% and in open interventions such as laparotomy and amputation, 17.6%. The gloves that were tested without surgery after putting them on, taking them off and transporting them, showed no damage. Of the 50 unused pairs of gloves tested from each of the three different manufacturers (batch testing of 300 gloves in total with 100 gloves per manufacturer), only one glove had a lesion at the inner wrist (0.3%). The damage was smaller than 1 mm. Thus, all were within the required AQL of 0.65. Procedures or equipment that could cause damage to gloves are listed in Table 4.

### 3.4. Size and Localization of the Glove Tears

**Figure 1:**
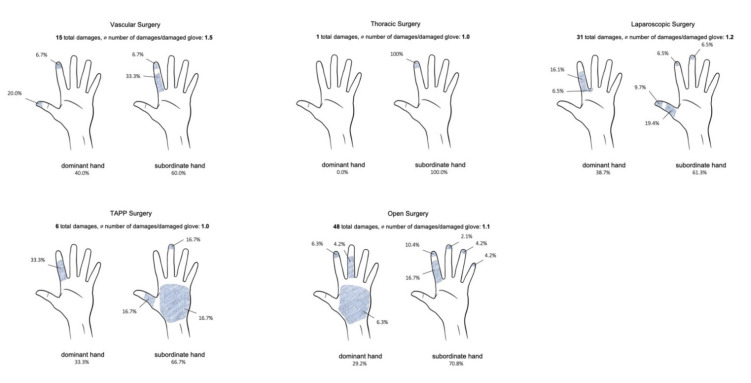
Distributions of damages with frequencies in % and number of damages per damaged glove according to investigated group of surgery.

**Figure 2:**
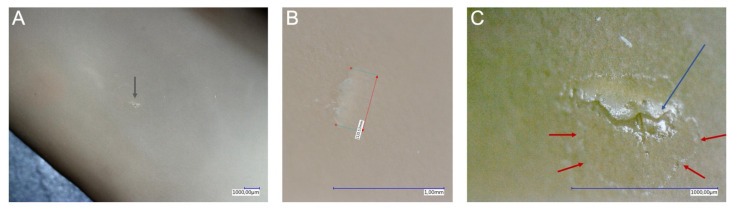
A typical microlesion of the glove as it occurs during endoscopic procedures (TAPP) under the digital microscope. (**A**) A microlesion with 20× magnification (grey arrow). (**B**) The lesion at 150× magnification and corresponding measurement of size. (**C**) The lesion at 200× depth magnification; the blue arrow marks the actual crack, and the red arrows mark the water leakage area as evidence of the pervasiveness of the lesion.

For a detailed analysis, see Table 3, Table 5 and Figure 1. In OS, 70.8% of the damage was in the subordinate hand, especially in the index finger area (16.7%) and its tip (10.4%); the dominant hand was affected in 29.2% of cases. The lesions during VS were also mainly located on the subordinate hand with 60.0%, and 33.3% of the lesions were in the index finger area. On the dominant hand, especially the tip of the thumb was damaged in 20.0% of the cases. The subordinate hand was 100.0% affected during TS. During LS, the subordinate hand was also damaged with 61.3%; 19.4% of the lesions affected the thumb and 9.7% the tip of the thumb. In comparison, 66.7% of TAPP procedures also showed the most damage to the subordinate hand, with 16.7% of the damage occurring in the thumb, middle fingertip and palm. In OS, most lesions (68.7%) showed a size of 1 mm, 10.4% were 2 mm, and 2.1% were 10 mm and were on the dominant hand. In VS, 73.3% of lesions were 1 mm in size, and 13.3% were 7 mm in size and were located on the subordinate hand. In TS, the size of the tears was 1 mm in 100% of the cases. In LS procedures, 1 mm (64.5%) lesions were also found in the majority of cases, while 2 and 3 mm lesions were found in 9.7% of cases. In the TAPP group, 100% of the damages were 1 mm in size, and 66.7% of the damages were in the subordinate hand. Figure 2 shows a typical microperforation during endoscopic procedures (TAPP); in the largest magnification, the area of water leakage can be seen in this microlesion. Larger damages could not be detected here.

## 4. Discussion

The use of gloves during surgical interventions is a generally accepted standard. The susceptibility to intraoperative damage during soft tissue surgery has been poorly investigated up to the present. In contrast, some studies of orthopaedic surgery have given rise to disturbing results [15,16]. 

### 4.1. Tears and Double Gloving

The majority of lesions are not noticed during surgeries, as shown by our investigation, in which even larger damages of 5 mm and more went unnoticed by the surgeon. A possible reason for the failed recognition of such large damages is discussed in detail in “Limitations”. However, when a lesion goes unnoticed, a realistic assessment of the risk for infection of the patient and the surgeon is not possible. Ideally, this situation should be prevented. In the present study, it was shown that even in soft tissue surgery, there were unexpectedly high numbers of lesions in gloves, which could endanger surgeons and patients if gloves became contaminated by bacteria during surgery [17]. Therefore, the use of so-called double-gloving is recommended in the literature [7] and by governmental institutions [3,18]. The benefits of double gloving have been investigated [13], but the percentage of surgeons who follow this counsel has not been conclusively clarified [19]. Reduced sense of tactile sensitivity or inexperienced handling of double gloving are cited as reasons not to follow this recommendation [20]. Even though soft tissue surgery appears to have little mechanical stress, the frictional forces generated during surgical knotting, for example, are probably sufficient to trigger lesions in gloves [21]. Often only the protective function of the glove towards the patient is mentioned, but the protection of the surgical team from pathogens of the patient is an equally important factor, and especially since most lesions take place unnoticed, double gloving should not be omitted from this point of view either [22]. The thickness of a glove seems to have a direct influence on the perforation rate, as it was shown that thicker gloves have significantly less damage compared to thinner gloves [23]. Thus, double gloving could be used to achieve a thickening of the gloves [24]. A simultaneous injury of the inner and outer glove is not described in the literature so far and could thus increase intraoperative safety; however, there could be unclear frictional forces between the latex layers. [24]. If double-gloving is chosen, the literature sees an advantage in the use of indicator gloves, which can give an early indication of glove lesions [13]. Given the rate of lesions detected in this study, double gloving is a procedure that should be strongly reconsidered. According to the results of this study, an alternative could be specialised gloves with corresponding strengthening in the affected damaged areas of the gloves. 

Furthermore, a planned glove change should be performed before handling sterile materials such as hernia mesh or vascular prostheses. Sterility of the extraneous materials is an absolute necessity, as possible bacterial contamination cannot be controlled by the body′s immunological system. Patients with immunosuppression in particular are at increased risk of graft infection [25]. The highest care should be taken perioperatively, as most infections are due to the surgical treatment [25]. Therefore, perioperative infection must be prevented, as the consequences for the patient are often severe [26]. A planned glove change could prevent possible unintentional contamination. This is also mentioned in the literature; Kobayashi et al. and Kim et al. also recommend changing gloves, especially after long duration of wear or before handling sterile materials to prevent infections during surgical interventions [27,28].

### 4.2. Tear Locations on Gloves

There are only limited data in the literature regarding the location of tears in gloves during soft tissue surgery. In orthopaedic and trauma surgeries such as total joint arthroplasty or internal fixation of bone fractures, lesions were primarily described in the area of the index finger of the dominant hand [15,29,30]. In the presented study most of the damage occurred at the subordinate hand during soft tissue surgical procedures. There was no difference between open and endoscopic surgeries regarding the location of the lesions. The index finger and thumb were most frequently affected. The localization of the damage is consistent with the published data on arthroscopic interventions, where the subordinate hand was also primarily affected [8]. When examining the data on laparoscopy in this study as well as on arthroscopy from a previous study [15], the thumb of the subordinate hand was particularly susceptible to damage in both of the procedures. In these minimally-invasive procedures, instruments and techniques used could be the deciding factor. The handling of the instruments could also play a role, since the instrument is usually carried by the dominant hand, which is thus protected. The subordinate hand carries out all other tasks, which increases the danger that the glove on the subordinate hand is damaged by the instrument that is carried in the dominant hand [31,32]. It should be noted that different working methods, differences in hand strength and skin hardness could have an impact on the damage pattern of gloves, as could the differences in glove manufacture. By using different manufacturers and studying surgeons of all genders with different surgical methods and physical conditions, a cross section of those factors was taken into account.

### 4.3. Size of Tears

In this study the majority of tear size were about 1 mm or less as far as they were detectable with the water tightening test. In other surgical fields as well, microlesions in gloves play an important role in both minimally invasive and open surgery [33]. Probably the lesion rate is even higher than assessed by the water tightness test, but the question of the relevance of detecting perforations of less than one millimetre has not been conclusively clarified [34]. The term microperforation is not uniformly used [14,30]. Based on this study, a size of less than or equal to one millimetre is suggested as the definition. Damages larger than 1 mm were mainly found in open surgery. It should be noted that extremity amputations were included in this group. This might have contributed to the increase in size of the lesions. However, larger tears up to 4 mm (6.5%) could also be found during endoscopic procedures. One reason could be that endoscopic procedures may be considered more invasive than arthroscopy, for example [8]. 

### 4.4. Lesion Rate and Surgery Duration

In this study, there was no significant correlation between the duration of surgery (wear time), the type of surgery and the lesion rate. This result is broadly consistent with other studies that have examined surgery time and damage rate [33]. In contrast, especially with mechanically demanding interventions, such as hip or knee endoprostheses or endoprosthetic revision procedures, it was shown that over time, due to accumulation of stress factors, mechanical damage increases in the course of the surgery [14,15]. However, the surgery duration of the majority of surgeries in this study was shorter than the threshold of 90 min, after which a glove exchange is recommended [14]. Hence the number of medium and long-term surgeries was underrepresented, thus potentially contributing to the failure to detect a correlation between wear time and lesion rate. Wear time is important, as apart from strong mechanical forces, repeated stress over a period of time—point loads in particular—can lead to damage to gloves. These stressors are inadequately reflected in the standardization and testing of gloves. 

### 4.5. Methods for Detecting Glove Damages and Standards

In the currently valid standards for testing surgical gloves for tightness, only the water tightness test is required [9,10,11]. Of course, this test is well suited for testing larger samples in industrial production, but it does not sufficiently reflect the risk potential of impermeable gloves. It has already been shown in the literature that this test method is not suitable for detecting microperforations [24]. While condoms are standardly tested for microperforations [12], this is not the standard procedure for surgical gloves. Test procedures based on electric current and conductivity would offer a much better resolution for the detection of microlesions.

## 5. Limitations of the Study

One limitation of the study is that only the gloves of the main surgeon were tested and not those of the whole team. Through the laboratory analysis it was not possible to differentiate whether the lesions were caused by the production process or during the surgery. Not all gloves were microscopically screened for damage, so further damage may have remained undetected. The impact on the postoperative wound infection rate as well as possible infections of the medical staff were not investigated. It was also surprising that relatively large lesions of 5 mm and more went unnoticed intra-surgically. Here we cannot rule out that smaller lesions that occurred intra-surgically were enlarged during the process of removing the gloves. 

## 6. Conclusions

The rate of glove damage in soft tissue surgery and especially in minimally invasive interventions seems relatively high. The protection of the patient but especially of the medical team could be unknowingly compromised as glove lesions and especially microlesions go undetected intraoperatively. The lesion rate seems to be independent of the duration of surgery. Based on the study, double gloving is strongly recommended even for procedures with low mechanical stress. Special glove exchange algorithms should be developed and applied. Gloves should be changed in particular before using sterile materials such as hernia mesh or vascular prostheses. 

## Figures and Tables

**Table 1:** Descriptive analysis of surgery and patient data.

	Vascular	Thorax	Laparoscopy	TAPP	Open	*p*-Values
Patient Data						
Number of patients recruited (*n*)	53	11	106	34	244	
BMI in kg/m^2^ (M ± SD; MD (range))	28.0 ± 6.0 27.0 (17.8–50.0)	30.3 ± 6.0 29.7 (22.1–38.6)	27.7 ± 6.2 26.0 (18.4–57.7)	26.0 ± 3.9 26.0 (20.3–36.3)	26.8 ± 4.7 26.6 (12.9–42.2)	0.185 (^)
Age in years (M ± SD; MD (range))	65.5 ± 14.8 68.0 (0–84) ^b^	61.1 ± 7.0 59.0 (51–74)	55.2 ± 19.8 56.5 (16–87) ^a,b^	61.1 ± 17.4 61.5 (18–92)	63.3 ± 17.2 67.0 (0–95) ^a^	0.003 (^)
Glove size (M ± SD; MD (range))	7.0 ± 0.5 7.0 (6.0–8.0) ^a,b,c^	7.6 ± 0.5 8.0 (6.5–8.0) ^c^	7.4 ± 0.6 7.5 (6.5–8.0) ^b^	7.3 ± 0.5 7.5 (6.5–8.0)	7.3 ± 0.5 7.5 (6.5–8.0) ^a^	0.000 (^)
Duration of use in minutes (M ± SD; MD (range))	100.4 ± 55.9 95.0 (19–241) ^a^	100.7 ± 61.6 103.0 (25–208)	83.7 ± 53.3 65.0 (27–307)	58.1 ± 27.0 52.5 (20–155) ^a^	95.0 ± 78.0 73.0 (2–397)	0.019 (^)

(^) Kruskal–Wallis test; (a,b,c) same small letters identify significant differences (*p* < 0.05).

**Table 2:** Overview of the individual operations of the different groups.

Group	Type of Surgery
Vascular (VS)	Thrombendarteriectomy, open abdominal aortic aneurysm repair, thoracic inlet syndrome, femoropopliteal bypass, shunt operations, carotid artery surgery, vascular graft implantation
Thorax (TS)	Pulmonary (partial) resections, thoracic hematoma evacuation, tracheotomies, pulmonary decortication, video-assisted thoracoscopy (VATS), thoracotomy (gastric pull-up)
Laparoscopy (LS)	Explorative laparoscopy, liver biopsy, scheduled peritoneal lavage, adhesiolysis, fundoplication, suturing gastric ulcer, gastric bypass, gastrectomy, cholecystectomy, intra-peritoneal onlay mesh technique (IPOM), appendectomy, colostomy, ileocecal resection, hemicolectomy, sigmoid resection, low anterior rectal resection.
TAPP	Transabdominal preperitoneal patch technique
Open (OS)	Amputation (various limbs), explorative laparotomy, parathyroidectomy, thyroidectomy, oesophageal resection, gastrectomy, PEG (percutaneous endoscopic gastrostomy), gastrostomy or jejunostomy by Witzel’s method, liver resection, cholecystectomy, pancreas resection, pancreatic drainage operation, pancreas and kidney transplantation, duodenum partial resection, cystojejunostomy, splenectomy, small bowel resection, ileostomy reversal, colorectal surgery (hemicolectomy, sigmoid resection, Hartmann-procedure, transverse colostomy rectum resection, rectopexy), pilonidal sinus surgery, excision of atheroma, excision of perianal abscess, haemorrhoidectomy, lymph node biopsy

**Table 3:** Statistical analysis of surgical data with regard to the occurrence of glove damage of the entire surgery. (*) Total comparison of all 5 groups using (^) Kruskal–Wallis test or (+) chi-square test differences.

	Vascular	Thorax	Laparoscopy	TAPP	Open	
OP-Specific Data						*p*-Value *
Total number of gloves used (*n*)	106	22	212	68	488	896 overall
Number of operations without damaged gloves (*n*, (%)) with damaged gloves (*n*, (%))	42 (79.2) 11 (20.8)	10 (90.9) 1 (9.1)	83 (78.3) 23 (21.7)	28 (82.4) 6 (17.6)	201 (82.4) 43 (17.6)	0.800 +
Number of surgical gloves undamaged (*n*, (%)) damaged (*n*, (%))	94 (88.7) 12 (11.3)	21 (95.5) 1 (4.5)	187 (88.2) 25 (11.8)	62 (91.2) 6 (8.8)	444 (91.0) 44 (9.0)	0.776 ^
Average number of damages per glove, damaged gloves only (M ± SD; MD (range))	1.5 ± 0.7 1 (1–3)	1.0 1 (1)	1.2 ± 0.6 1 (1–3)	1.0 1 (1)	1.1 ± 0.4 1 (1–3)	0.249 ^

(^) Kruskal–Wallis test, (+) chi-square test, (*) Total comparison of all 5 groups.

**Table 4:** Factors associated with the risk of glove damage.

Group	Factors
Vascular (VS)	Needles and scalpels, surgical instruments *, vascular grafts, surgical sutures and knotting, spot tension
Thorax (TS)	Surgical instruments *, needles and scalpels, grafts, surgical sutures and knotting, spot tension
Laparoscopy (LS)	Rotating instruments, sharp-edged instruments, mesh grafts, needles and scalpels, surgical sutures and knotting, traction using the subordinate hand, surgical instruments *
TAPP	Surgical instruments *, rotating instruments, mesh grafts, surgical sutures and knotting, needles and scalpels, traction using the subordinate hand
Open (OS)	Surgical instruments *, sharpened bone edges, grafts, needles and scalpels, implant handling, surgical sutures and knotting, spot tension

* Surgical instruments (for example: scissors, needle holders, or forceps).

**Table 5:** Percentage occurrence of certain sizes of damage in relation to the total number of damages.

	Vascular		Thorax		Laparoscopy		TAPP		Open		
Total number of damages	15		1		31		6		48		
mm	DH	SH	DH	SH	DH	SH	DH	SH	DH	SH	
1	40.0	33.0	0.0	100.0	22.6	41.9	33.3	66.7	20.8	47.9	
2	0.0	0.0	0.0	0.0	6.5	9.7	0.0	0.0	2.1	8.3	
3	0.0	6.7	0.0	0.0	3.2	9.7	0.0	0.0	2.1	6.3	
4	0.0	0.0	0.0	0.0	6.5	0.0	0.0	0.0	2.1	6.3	
5	0.0	0.0	0.0	0.0	0.0	0.0	0.0	0.0	0.0	2.1	
6	0.0	0.0	0.0	0.0	0.0	0.0	0.0	0.0	0.0	0.0	
7	0.0	13.3	0.0	0.0	0.0	0.0	0.0	0.0	0.0	0.0	
8	0.0	0.0	0.0	0.0	0.0	0.0	0.0	0.0	0.0	0.0	
9	0.0	0.0	0.0	0.0	0.0	0.0	0.0	0.0	0.0	0.0	
10	0.0	0.0	0.0	0.0	0.0	0.0	0.0	0.0	2.1	0.0	
>10	0.0	6.7	0.0	0.0	0.0	0.0	0.0	0.0	0.0	0.0	

The total number of damages may differ from the total number of damaged gloves because in some cases several damages per glove were found. DH: dominant hand, SH: subordinate hand. Data in %.

## Data Availability

Data are stored at the Orthopaedic clinic and policlinic, University medical centre Rostock and available on request from the corresponding author.
